# Supplementary material for: Atomic-scale observation of geometric reconstruction in a fluorine-intercalated infinite layer nickelate superlattice
Source: Nat Commun. 2025 Apr 6;16:3277. doi: 10.1038/s41467-025-58646-0 (PMC11972391; doi:10.1038/s41467-025-58646-0)
Supplement: Supplementary file 1 — Supplementary Information [file 41467_2025_58646_MOESM1_ESM.pdf]

# Supplementary information for
# Atomic-scale observation of geometry reconstruction in a fluorine-intercalated infinite layer nickelate superlattice

Chao Yang[1*,†], Roberto A. Ortiz[1, †], Hongguang Wang[1], Wilfried Sigle[1], Kelvin Anggara[1], Eva Benckiser[1], Bernhard Keimer[1], Peter A. van Aken[1]

[1]Max Planck Institute for Solid State Research, Stuttgart, 70569, Germany

*Corresponding author: c.yang@fkf.mpg.de

†Equal contribution

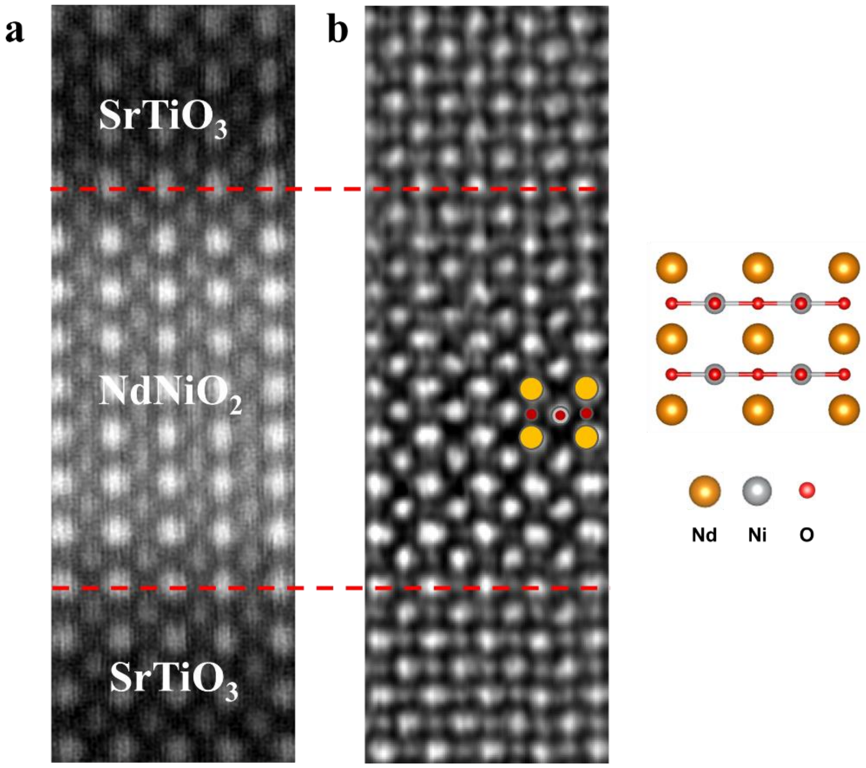


Figure S1: Atomic structure of an $8NdNiO_2/4SrTiO_3$ superlattice film. Reconstructed (a) ADF and (b) iCoM images of the $NdNiO_2/SrTiO_3$ superlattice sample. The infinite layer structure model is shown on the right side.

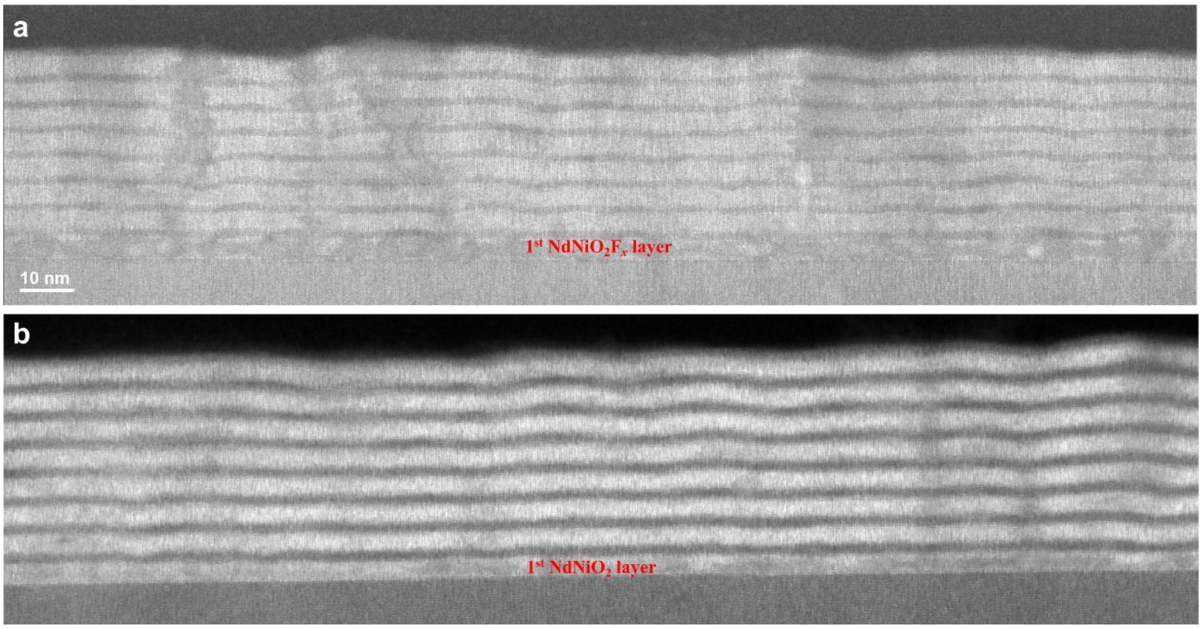


Figure S2: Microstructure of $8NdNiO_2F_x/4SrTiO_3$ and $8NdNiO_2/4SrTiO_3$ superlattices. Overview HAADF images of (a) $8NdNiO_2F_x/4SrTiO_3$ superlattice and (b) $8NdNiO_2/4SrTiO_3$ superlattice.

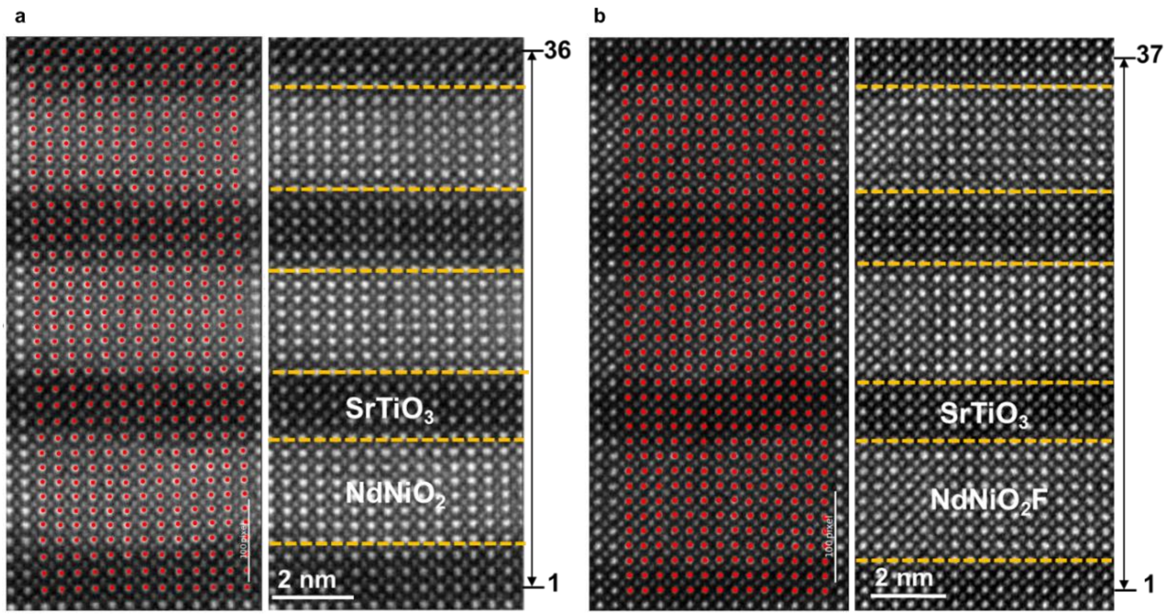


Figure S3: Atomic structure and Nd column fitting maps of 8$NdNiO_2$/4$SrTiO_3$ and 8$NdNiO_2F_x$/4$SrTiO_3$ superlattices. (a) HAADF image of the 8$NdNiO_2$/4$SrTiO_3$ superlattice sample, together with an overlay plot of the marked Nd columns. (b) HAADF image of the 8$NdNiO_2F_x$/4$SrTiO_3$ superlattice sample, together with an overlay plot of the marked Nd columns.

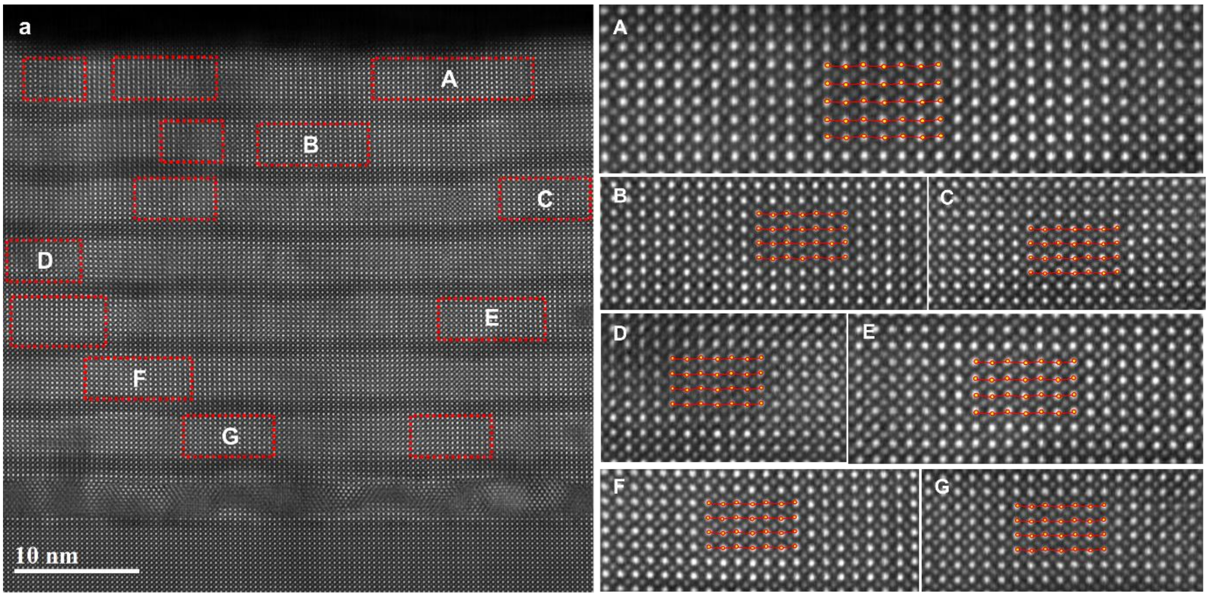


Figure S4: Atomic structure of a F-intercalated $NdNiO_2/SrTiO_3$ superlattice. (a) An overview HAADF image of the F-intercalated $NdNiO_2/SrTiO_3$ superlattice sample. The red dashed boxes highlight the region with pronounced zigzag arrangement of Nd columns. The magnified A-G regions are shown on the right. Yellow dot and red lines mark the zigzag arrangement of Nd atoms.

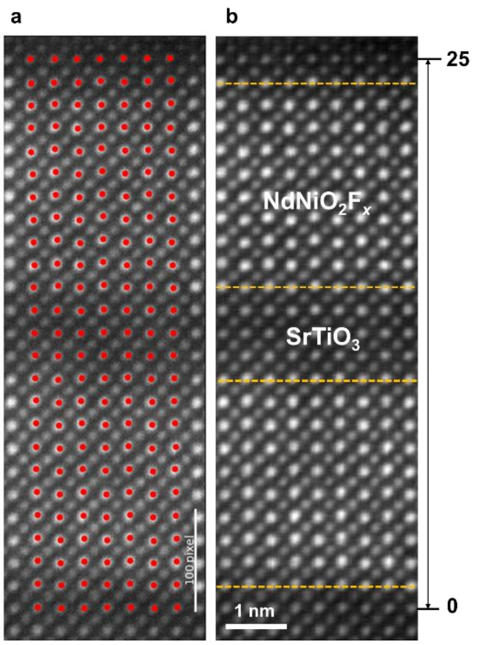


Figure S5: Atomic structure and Nd column fitting map of an 8$NdNiO_2F_x$/4$SrTiO_3$ superlattice. (b) HAADF image of the 8$NdNiO_2F_x$/4$SrTiO_3$ superlattice sample, together with (a) an overlay plot of the marked Nd columns.

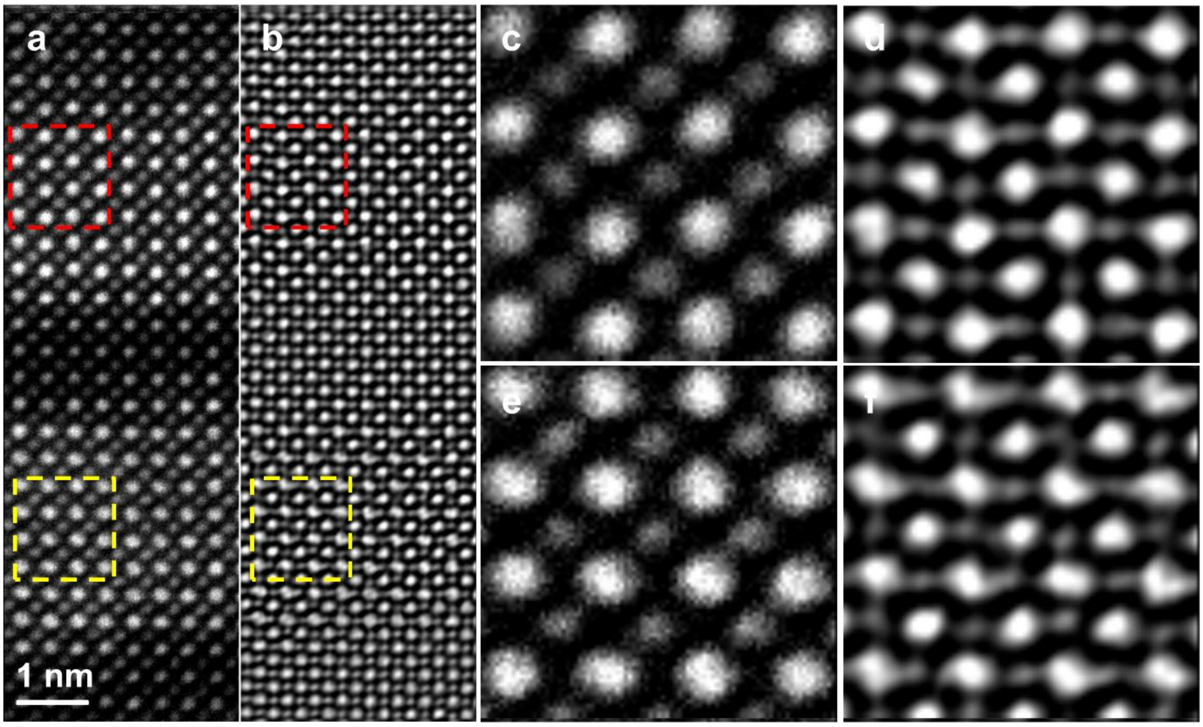


Figure S6: Atomic structure of domains in F-intercalated $NdNiO_2$ layers. Reconstructed (a) ADF and (b) iCoM images of the F-intercalated $NdNiO_2/SrTiO_3$ superlattice sample. The magnified (c) ADF and (d) iCoM images of the region marked by the red dashed boxes in (a) and (b) show the domain with visible zigzag arrangement of Nd atoms. The magnified (e) ADF and (f) iCoM images of the region marked by the yellow dashed boxes in (a) and (b) show the domain with invisible zigzag arrangement of Nd atoms.

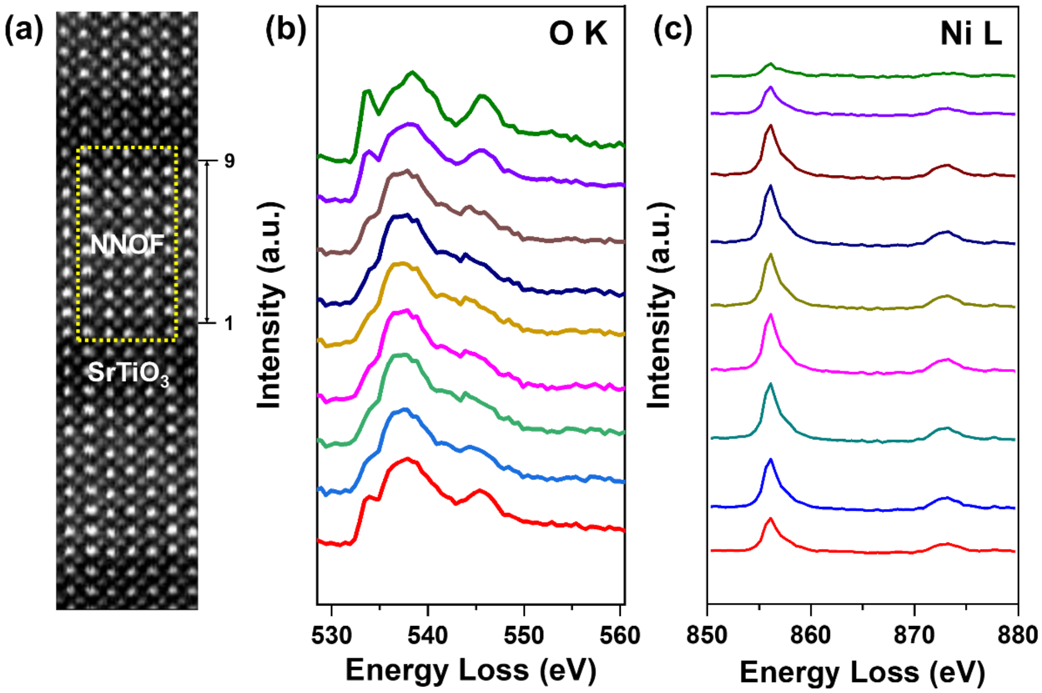


Figure S7: EELS measurements of O and Ni edges. (a) HAADF image of the region for EELS measurements. EELS spectra of (b) O K edges and (c) Ni L edges extracted from the region marked with yellow dashed boxes in (a).

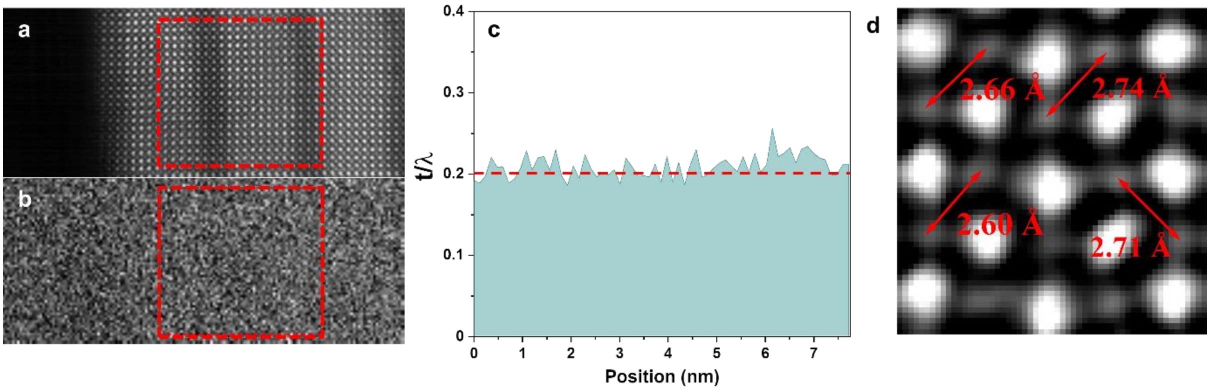


Figure S8: Measurement of sample thickness. (a) The ADF image of the region for the EELS measurement and (b) the corresponding t/λ map. (c) The averaged t/λ line profile from the region marked by the red dashed box in (b). t is the sample thickness and λ is the local inelastic mean free path. (d) The iCoM image with a zigzag arrangement of the Nd atoms. The red lines and values mark the distances of the neighboring anions.

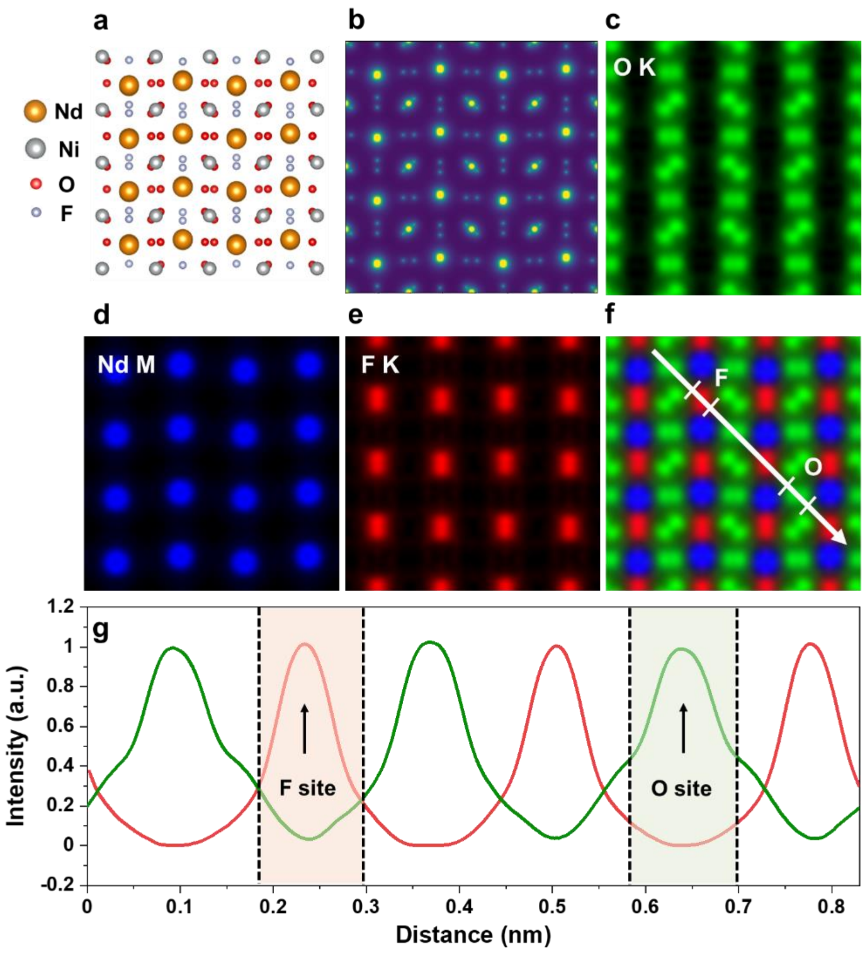


Figure S9: Simulated elemental EELS Maps. (a) Structural model of $NdNiO_2F$ with a thickness of 15 nm and (b) the corresponding projected potential image. Simulated EELS elemental maps of (c) O K, (d) Nd M, and (e) F K. (f) Composite elemental map. (g) Line profile extracted from the region marked with a white arrow in (f). Red and green boxes mark the regions for F and O signal intensity calculation, respectively.

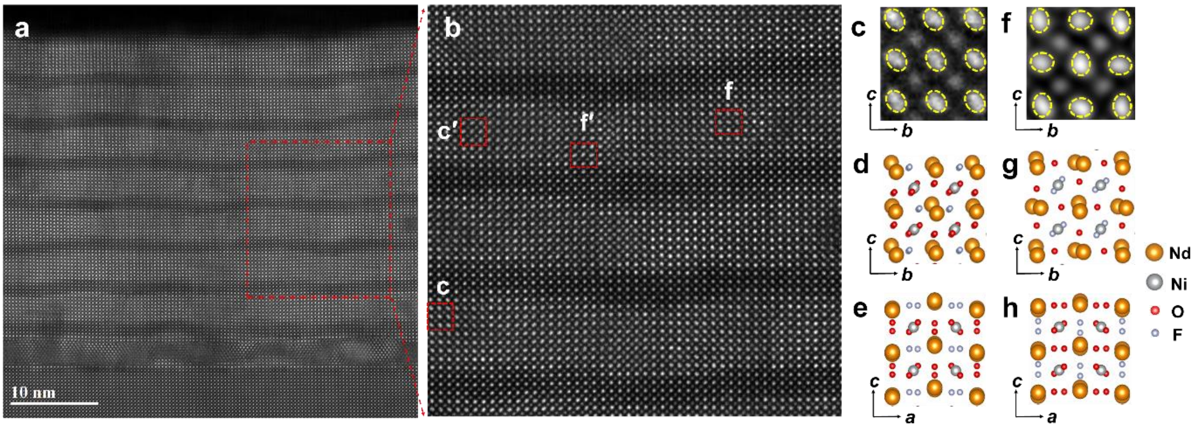


Figure S10: Observation of elliptical Nd columns. (a) Overview and (b) enlarged HAADF images of the $NdNiO_2F_x/SrTiO_3$ superlattice sample. (c) HAADF image of the region with elliptical Nd columns pointing in the same direction. The corresponding structure models with F intercalation at apical sites for viewing directions [100] in (d) and [010] in (e), respectively. (f) HAADF image of the region with perpendicular elliptical Nd columns. The corresponding structure models with F intercalation at basal sites for the viewing directions (g) [100] and (h) [010], respectively. The red boxes labeled c, c′, f, and f′ in (b) indicate the analogous enlarged regions of (c) and (f).

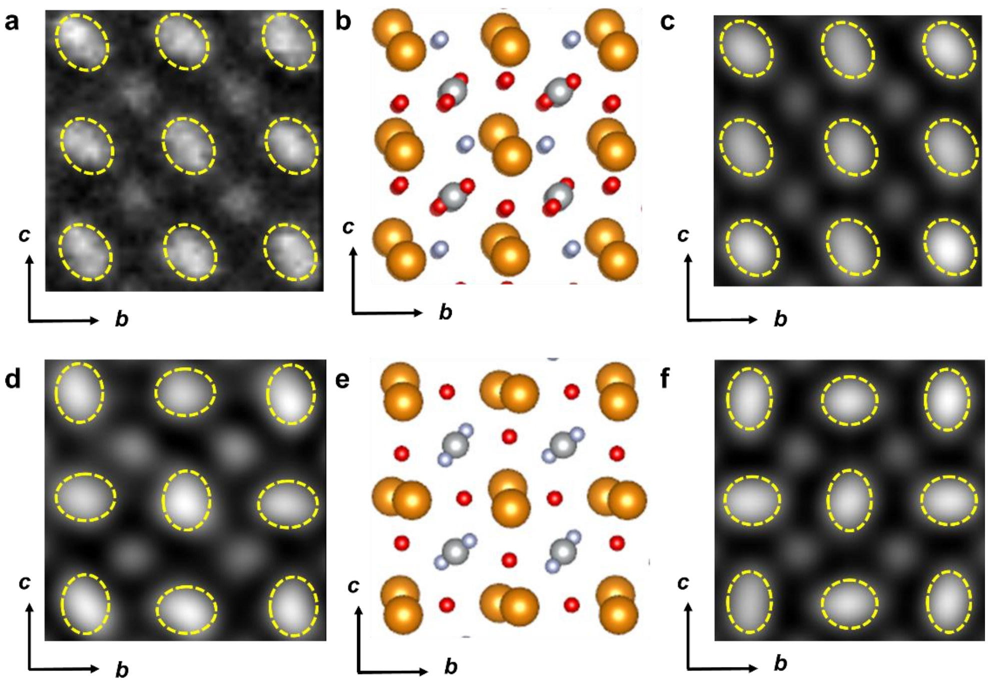


Figure S11: The effects of F intercalation locations on the elliptical shape of Nd columns. (a) HAADF image of the region with elliptical Nd columns pointing in the same direction. (b) The corresponding structure model with F intercalation at apical sites for viewing direction [100] and (c) the simulated HAADF image. (d) HAADF image of the region with perpendicular elliptical Nd columns. (e) The corresponding structure models with F intercalation at basal sites for the viewing direction [100] and (f) the simulated HAADF image. Elliptical Nd columns are highlighted with yellow dashed ellipses.

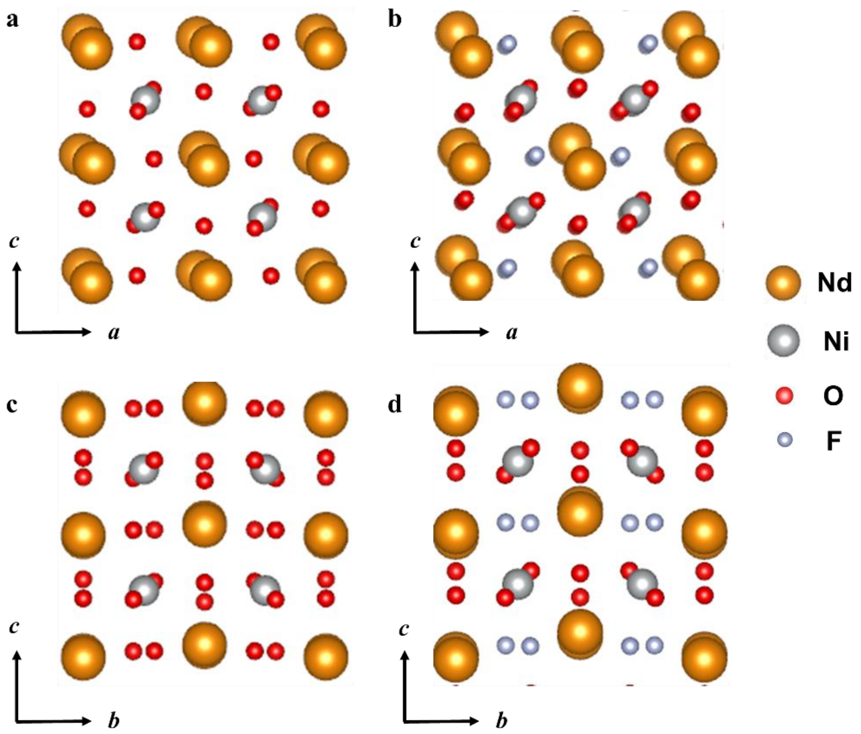


Figure S12: Structural models of $NdNiO_3$ and oxyfluoride $NdNiO_2F$ phases. Structural models of the perovskite phase of $NdNiO_3$ from the directions of view (a) [010] and (c) [100]. Structural models of the oxyfluoride $NdNiO_2F$ with the F-ions intercalated at the apical sites from viewing directions (b) [010] and (d) [100].

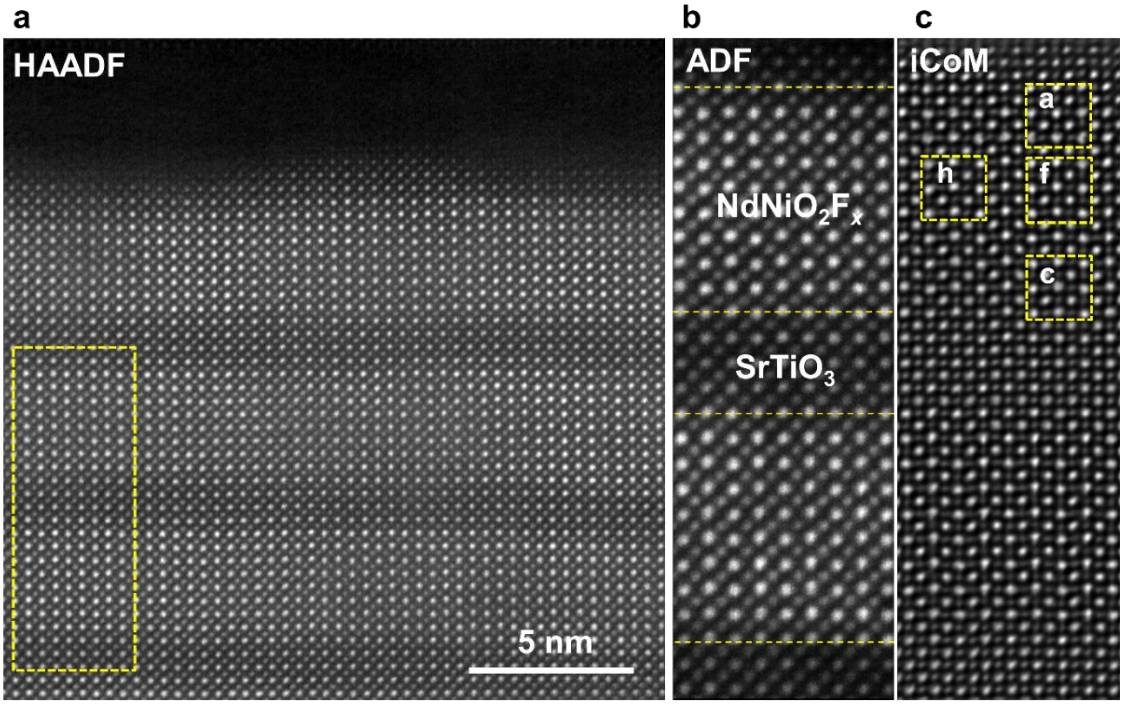


Figure S13: Observation of anion sublattice. (a) Overview HAADF image of the $NdNiO_2F_x/SrTiO_3$ superlattice sample. The yellow dashed box marks the region for the 4D-STEM measurement. Reconstructed (b) ADF and (c) iCoM images for the enlarged region. The yellow dashed boxes in (c) show the regions of (a, c, f, and h) in Figure 5 in the main text.

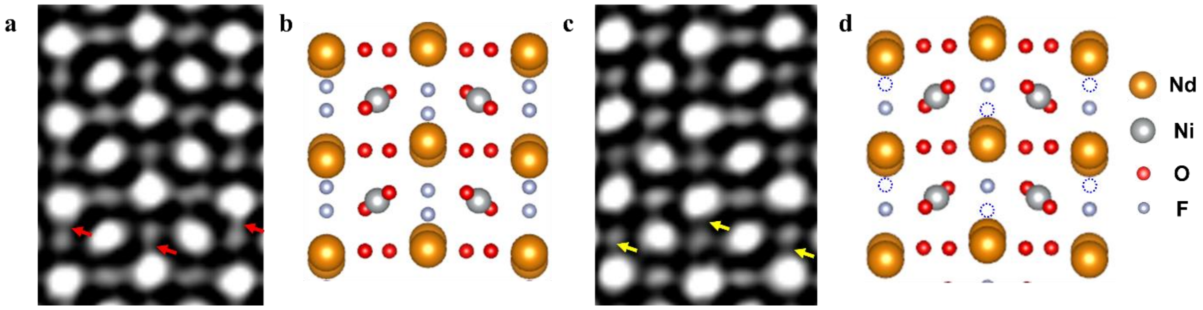


Figure S14: Anion sublattice with F intercalation at basal sites. (a) Reconstructed iCoM image with visible contrast between the basal anions and the Nd columns, marked with red arrows. (b) The corresponding structure model where the intercalation of F ions at the basal sites induces a strong polyhedral distortion. (c) Reconstructed iCoM image without clear contrast between the basal anions and the Nd columns, marked with yellow arrows. (d) The structure model with partial intercalation of F ions at the basal sites, manifested by a missing basal anion, marked with blue dashed circles.

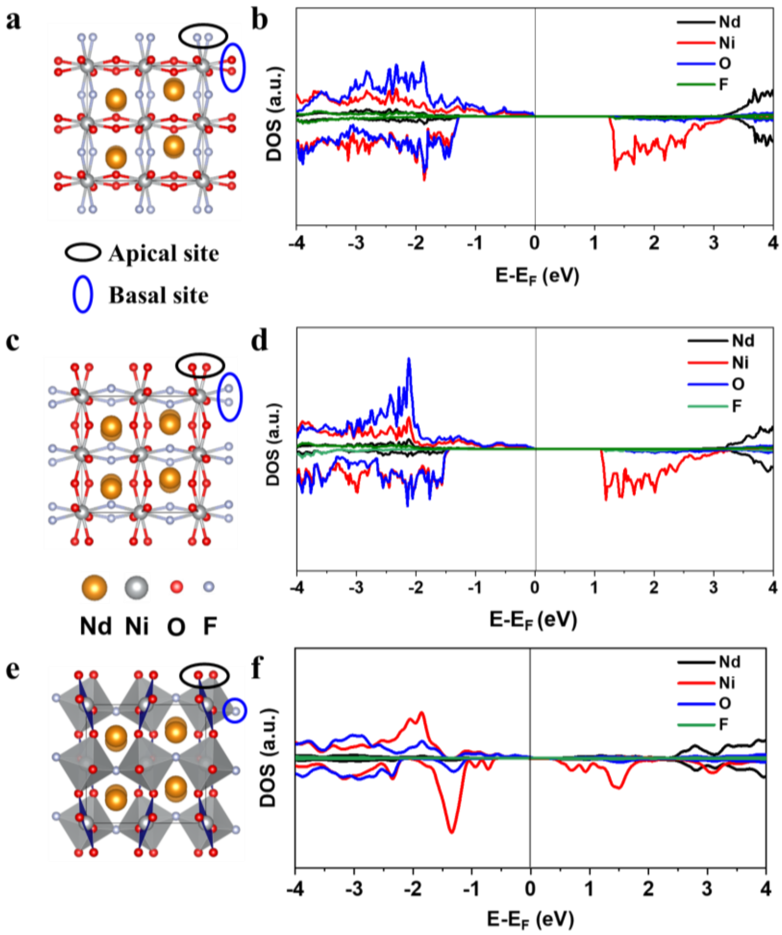


Figure S15: Density of states for different configurations of F-intercalated $NdNiO_2$ layers. Structure models with (a) the intercalation of F ions at the apical sites, (c) the intercalation of F ions at the basal sites, and (e) the partial intercalation of F ions at the basal sites, corresponding to the experimental structures shown in Figures 5d, S9f, and 5k, respectively. The corresponding density of states are shown in (b), (d), and (f), respectively. The black lines indicate the Fermi level.

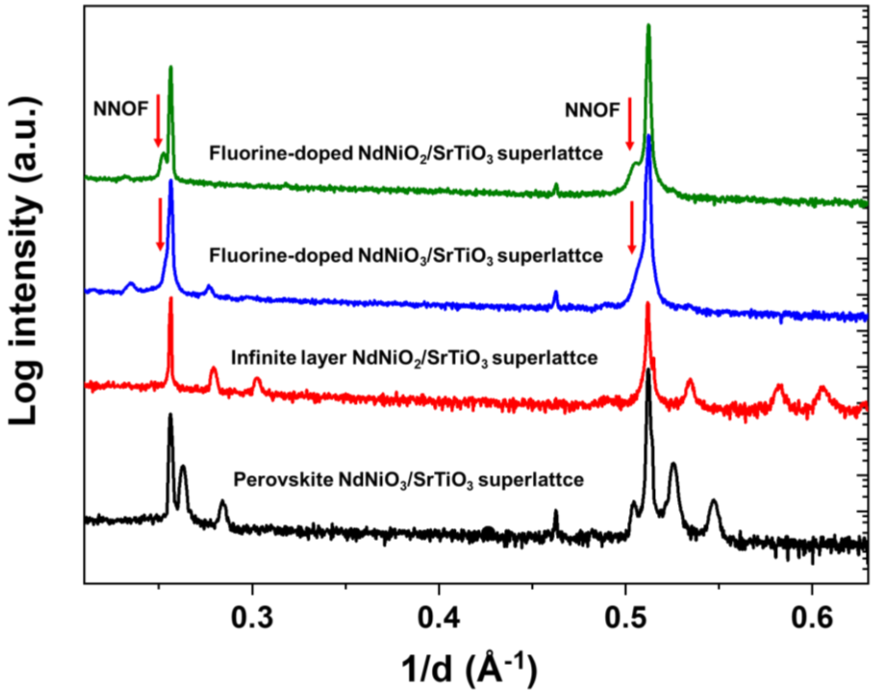


Figure S16: XRD results of the perovskite nickelate/$SrTiO_3$ superlattice, the infinite layer nickelate/$SrTiO_3$ superlattice, the fluorine-doped perovskite nickelate/$SrTiO_3$ superlattice, and the fluorine-doped infinite layer nickelate/$SrTiO_3$ superlattice samples. The fluorination of the perovskite nickelate/$SrTiO_3$ superlattice was carried out at 350°C for 168 hours. Fluorination of the infinite layer nickelate/$SrTiO_3$ superlattice was performed at 350°C for 24 hours. The red arrows indicate the XRD peaks of the $NdNiO_2F_x$ phase.
